# Real-world effectiveness of steroids in severe COVID-19: a retrospective cohort study

**DOI:** 10.1186/s12879-022-07750-3

**Published:** 2022-10-05

**Authors:** Wenjuan Wang, Luke B Snell, Davide Ferrari, Anna L Goodman, Nicholas M Price, Charles D Wolfe, Vasa Curcin, Jonathan D Edgeworth, Yanzhong Wang

**Affiliations:** 1 School of Population Health and Environmental Sciences, King’s College London, London, UK; 2 Centre for Clinical Infection and Diagnostics Research, School of Immunology and Microbial Sciences, King’s College London, London, UK; 3 Department of Infectious Diseases, Guy’s and St Thomas’ NHS Foundation Trust, London, UK; 4 NIHR Biomedical Research Centre, Guy’s and St Thomas’ NHS Foundation Trust, London, UK

**Keywords:** COVID-19, Steroids, Mortality, Retrospective cohort study

## Abstract

**Introduction:**

Randomised controlled trials have shown that steroids reduce the risk of dying in patients with severe Coronavirus disease 2019 (COVID-19), whilst many real-world studies have failed to replicate this result. We aim to investigate real-world effectiveness of steroids in severe COVID-19.

**Methods:**

Clinical, demographic, and viral genome data extracted from electronic patient record (EPR) was analysed from all SARS-CoV-2 RNA positive patients admitted with severe COVID-19, defined by hypoxia at presentation, between March 13th 2020 and May 27th 2021. Steroid treatment was measured by the number of prescription-days with dexamethasone, hydrocortisone, prednisolone or methylprednisolone. The association between steroid > 3 days treatment and disease outcome was explored using multivariable cox proportional hazards models with adjustment for confounders (including age, gender, ethnicity, co-morbidities and SARS-CoV-2 variant). The outcome was in-hospital mortality.

**Results:**

1100 severe COVID-19 cases were identified having crude hospital mortality of 15.3%. 793/1100 (72.1%) individuals were treated with steroids and 513/1100 (46.6%) received steroid ≤ 3 days. From the multivariate model, steroid > 3 days was associated with decreased hazard of in-hospital mortality (HR: 0.47 (95% CI: 0.31–0.72)).

**Conclusion:**

The protective effect of steroid treatment for severe COVID-19 reported in randomised clinical trials was replicated in this retrospective study of a large real-world cohort.

**Supplementary Information:**

The online version contains supplementary material available at 10.1186/s12879-022-07750-3.

## Background

Currently, steroids are the main treatment for severe coronavirus disease 2019 (COVID-19) infection [1], which has infected over 540million people and caused over 6million deaths worldwide [2]. The RECOVERY trial [3, 4] was the first randomised controlled trial to show that in patients hospitalized with COVID-19, the use of dexamethasone resulted in lower 28-day mortality among those who were receiving either invasive mechanical ventilation or oxygen alone but not among those receiving no respiratory support. Some meta-analyses have shown a benefit of steroids at preventing mortality [5, 6] and reducing need for mechanical ventilation [6]. However, other meta-anlysis from both observational studies and randomised controlled trials have shown conflicting results [7, 8].

A guideline was issued by WHO on use of dexamethasone and other corticosteroids (hydrocortisone or prednisone) for treatment of severe and critically unwell COVID-19 patients in September 2020 [9]. After the RECOVERY trial and WHO guidelines, the use of steroids changed from being used in ICU for some very severe patients, to more consistent use in patients admitted to hospital requiring oxygen. Our objective was to determine whether the effect of steroids on outcomes for severe COVID-19 patients reported in randomised trials is replicated in a large real-world cohort spanning the duration of the pandemic.

## Methods

### Population of interest and setting

Guy’s and St Thomas’ NHS Foundation Trust (GSTT) is a multi-site inner-city healthcare institution providing general and emergency services predominantly to the South London boroughs of Lambeth and Southwark. NHS is the National Health Service in the UK. The acute-admitting site (St Thomas’ Hospital) has an emergency department with a large critical care service. A second hospital site (Guy’s Hospital) provides elective surgery, haemato-oncology, renal transplantation and other specialist services. There are also several community sites providing dialysis, rehabilitation and long-term care. Only COVID-19 cases admitted through the emergency department (ED) during March 13th 2020 and May 27th 2021 were included in this study. Patients dying or being discharged in the first 24h were considered most likely to have reached study endpoint independent of any steroid effect and were excluded from the primary analysis.

### SARS-CoV-2 laboratory testing

GSTT has an on-site laboratory providing SARS-CoV-2 testing to all patients and hospital care workers (HCW). The policies and technologies employed for SARS-CoV-2 testing changed over time based on national and local screening guidance and improvements in diagnostics. Our laboratory began testing on 13th March 2020 with initial capacity for around 150 tests per day, before increasing to around 500 tests per day in late April during wave one, and up to 1000 tests per day during the second wave.

Assays used for the detection of SARS-CoV-2 RNA include PCR testing using Aus Diagnostics or by the Hologic Aptima SARS-CoV-2 Assay. Testing commenced during the first wave on 13th March 2020 limited to cases requiring admission or inpatients who had symptoms of fever or cough, as per national recommendation; guidance suggested cases who did not require admission should not be tested. Cases without laboratory confirmation of SARS-CoV-2 infection were not included.

### Definitions

Cases were identified by the first positive SARS-CoV-2 RNA test. The severe cases were measured by hypoxia upon admission to hospital. Cases were taken to be hypoxic if on admission they had oxygen saturations of < 94%, if they were recorded as requiring supplemental oxygen, or if the fraction of inspired oxygen was recorded as being greater than 0.21.

### Determination of SARS-CoV-2 lineage

Whole genome sequencing of residual samples from SARS-CoV-2 cases was performed using GridION (Oxford Nanopore Technology), using version 3 of the ARTIC protocol [10] and bioinformatics pipeline [11]. Samples were selected for sequencing if the corrected CT value was 33 or below, or the Hologic Aptima assay was above 1000 relative light units (RLU). During the first wave sequencing occurred between March 1st − 31st, whilst sequencing restarted in November 2020 and is ongoing. Lineage determination was performed using updated versions of pangolin 2.0 [12]. Samples were regarded as successfully sequenced if over 50% of the genome was recovered and if lineage assignment by pangolin was given with at least 50% confidence.

### Data sources, extraction and integration

Clinical, laboratory and demographic data for all cases with a laboratory reported SARS-CoV-2 PCR RNA test on nose and throat swabs or lower respiratory tract specimens were extracted from hospital electronic patient record (EPR) data sources using records closest to the test date (DXC Technology’s i.CM EPR, Philips IntelliVue Clinical Information Portfolio (ICIP) Critical Care, DXC Technology’s MedChart, e-Noting and Citrix Remote PACS - Sectra). Data was linked to the Index of Multiple Deprivation (IMD), with 1 denoting the least deprived areas, and 5 the most deprived ones. Age and sex were extracted from EPR. Self-reported ethnicity of cases was stratified to be White, BAME (Black, Asian and Minority Ethnic) and Unknown according to the 18 ONS categories of White (British, Irish, Gypsy and White-Other), Black (African, Caribbean, and Black-Other), Asian (Bangladeshi, Chinese, Indian, Pakistan, and Asian-Other), and Mixed/Other.

Comorbidities, medication history, and medicine data were extracted from the EPR and e-Noting using structured queries with corresponding dictionaries. Comorbidities were extracted from any of the databases covering the pathway of the cases from arrival in accident and emergency through inpatient general ward and critical care unit, where applicable, to hospital discharge or death. If a comorbidity was not recorded, we assume that it was not present. Cases were characterised as having/not having a past medical history of hypertension, cardiovascular disease (stroke, transient ischaemic attack, atrial fibrillation, congestive heart failure, ischaemic heart disease, peripheral artery disease or atherosclerotic disease), diabetes mellitus, chronic kidney disease, chronic respiratory disease (chronic obstructive pulmonary disease, asthma, bronchiectasis or pulmonary fibrosis) and neoplastic disease (solid tumours, haematological neoplasias or metastatic disease). Additionally, checks on free text data were performed by a cardiovascular clinician to ensure the information was accurate.

### Steroids

Steroid treatment was measured by number of prescription-days with dexamethasone, hydrocortisone, prednisolone or methylprednisolone. Duration of treatment with steroids was calculated as cumulative days throughout first hospital admission after the first SARS-CoV-2 PCR positive test through to discharge or death during that admission. Analysis for lengths of steroid use were conducted in multivariate model with steroid use ≤ 3 days versus steroid use > 3 days. The cut-off for the steroid treatment days were chosen according to the interquartile range of steroid-days (3 to 10 days) in RECOVERY trial. Sensitivity analysis was conducted with continuous steroid days as the variable input in the Cox proportional hazards model.

### Outcomes

The outcome was all-cause in-hospital mortality (WHO-COVID-19 Outcomes Scale 8), with patients still hospitalised at the end of the cohort considered censored.

### Statistical analysis

The general statistics were summarised with mean and standard deviation (SD) for continuous variables if the distribution is normal and median and interquartile range (IQR) if the distribution is non-normal. Count and percentages were used for categorical variables. For the comparisons of the cohort statistics with different lengths of steroid use days (< 3 days vs. ≥ 3 days), Kruskal-Walllis test was used for continuous variables and Chi-squared test for categorical variables. The reference significant level was set to be p < 0.05.

Cox proportional hazards models were used for time-to-event survival analysis in which the time was starting from hospital admission and events as the defined outcomes. Adjusted hazards ratios for the primary and secondary outcomes using Cox proportional hazards models were presented. The adjusted variables used in the model were selected via literature review [4] and clinical experts (Additional file Table A). Age, sex, Body Mass Index (BMI) > 30kg/m^2^, hypertension, cardiovascular disease, diabetes, respiratory disease, chronic kidney disease, sequenced SARS-CoV-2 variant and medications including steroids and tocilizumab/sarilumab were used as pre-defined covariates to adjust in multivariable models. As the distribution of steroid days is right skewed (steroid days ≥ 0), before modelling, the continuous steroid days were transformed with the log of steroid days plus one (log(steroid days + 1)). Missing values of the variant, BMI and ethnicity were imputed as a new category and cases with missing values in IMD were discarded. There were no missing values in other adjusted variables.

Data management was performed using SQL databases, with analysis carried out on the secure King’s Health Partners (KHP) Rosalind high-performance computer infrastructure [5] running Jupyter Notebook 6.0.3, R 3.6.3 and Python 3.7.6.

## Results

### Description of population, steroid use and outcomes

**Fig. 1:**
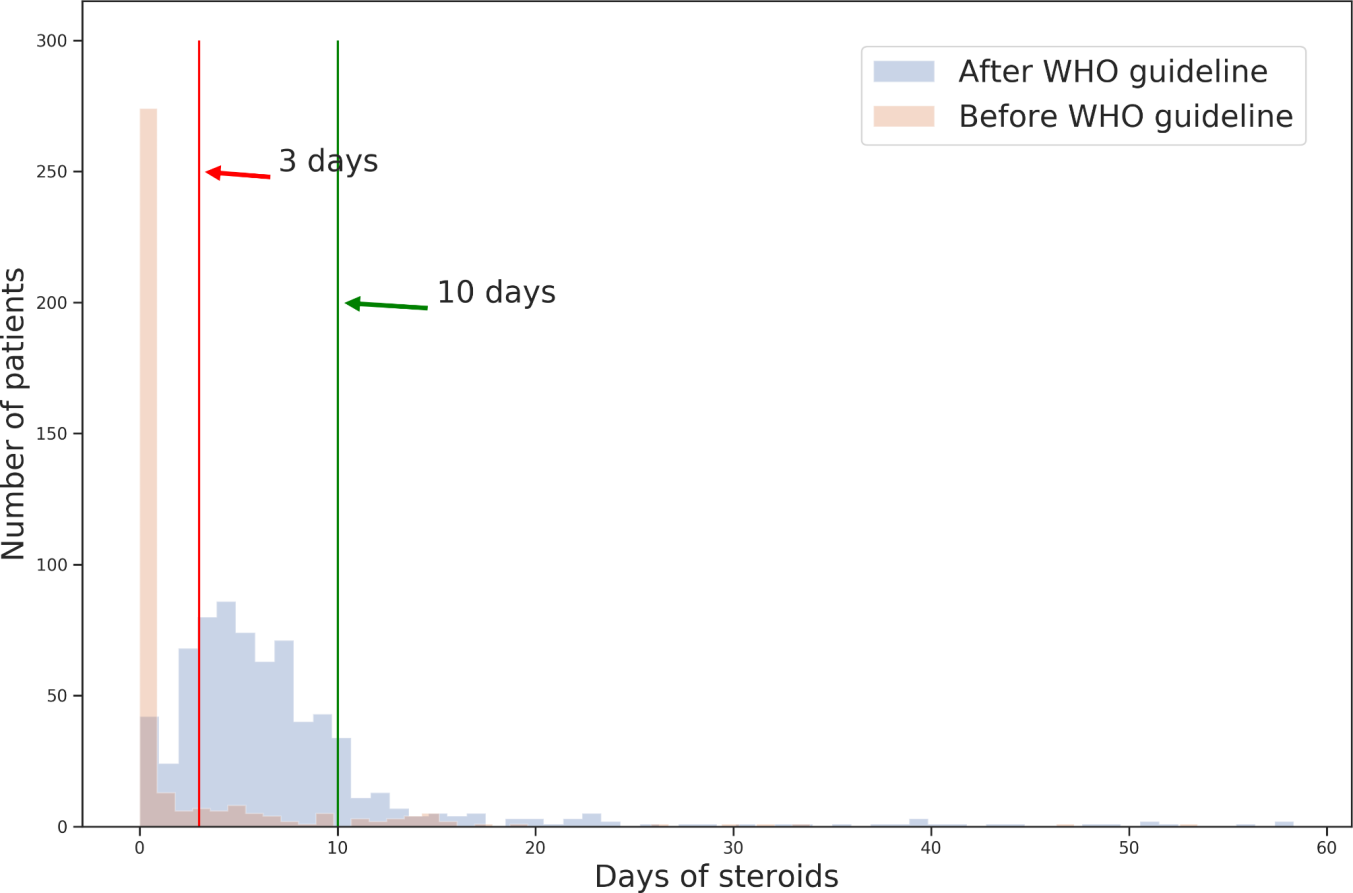
Frequency of steroid treatment-days for patients admitted before and after WHO guideline

1120 patients were identified with hypoxia on admission of which 1100 were included in the analysis after removal of 20 cases that stayed for less than 24h after admission. 23 cases with missing data in the IMD variable were imputed with median. In-hospital mortality of the whole cohort was 15.0% (Table1). 793/1100 (72.1%) individuals were treated with steroids (> 0 days) and the median of steroid days was 6.0, IQR [3,9]. Before the WHO guideline, only 96/366 (26.2%) patients were treated with steroids compared to 697/734 (95%) after the WHO guideline (Table2; Fig.1). Overall, steroids were used for a median of 0 days [IQR: 0.0,1.0] before the WHO guideline, and 5.5 days [IQR: 3.0,9.0] after WHO guideline. Before the WHO guideline, 17.2% patients had more than 3 days steroids and 7.9% more than 10 days, whilst after the WHO guidelines 71.4% had more than 3 days and 14.3% had more than 10 days (Table2).

**Table 1:** Characteristics for patient groups receiving different steroid treatment-days during their hospital admission

Steroids	Overall	≤ 3 days	> 3 days	P-value
n	1100	513 (46.6%)	587 (53.4%)	
Age, median [Q1,Q3]	63.0 [53.0,77.0]	62.0 [51.0,79.0]	64.0 [53.0,75.0]	0.552
Male, n (%)	624 (56.7)	290 (56.5)	334 (56.9)	0.950
Ethnicity, n (%)				0.035
White	436 (39.6)	183 (35.7)	253 (43.1)	
BAME	453 (41.2)	229 (44.6)	224 (38.2)	
Unknown	211 (19.2)	101 (19.7)	110 (18.7)	
BMI, n (%)				**0.003**
BMI ≤ 30	524 (47.6)	246 (48.0)	278 (47.4)	
BMI > 30	376 (34.2)	155 (30.2)	221 (37.6)	
BMI Unknown	200 (18.2)	112 (21.8)	88 (15.0)	
Cardiovascular, n (%)	286 (26.0)	132 (25.7)	154 (26.2)	0.903
Hypertension, n (%)	397 (36.1)	166 (32.4)	231 (39.4)	**0.019**
Diabetes, n (%)	316 (28.7)	142 (27.7)	174 (29.6)	0.515
Chronic respiratory disease, n (%)	190 (17.3)	80 (15.6)	110 (18.7)	0.195
Cancer, n (%)	57 (5.2)	22 (4.3)	35 (6.0)	0.266
Kidney disease, n (%)	135 (12.3)	62 (12.1)	73 (12.4)	0.933
HIV, n (%)	30 (2.7)	13 (2.5)	17 (2.9)	0.855
Transplant, n (%)	26 (2.4)	5 (1.0)	21 (3.6)	**0.008**
IMD Quintile, n (%)				0.566
1	264 (24.5)	116 (23.0)	148 (25.9)	
2	553 (51.3)	256 (50.7)	297 (51.9)	
3	159 (14.8)	81 (16.0)	78 (13.6)	
4	69 (6.4)	36 (7.1)	33 (5.8)	
5	32 (3.0)	16 (3.2)	16 (2.8)	
Tocilizumab or sarilumab, n (%)	12 (1.1)		12 (2.0)	**0.003**
Variant (%)				**< 0.001**
Alpha	211 (19.2)	53 (10.3)	158 (26.9)	
non-Alpha	383 (34.8)	306 (59.6)	77 (13.1)	
Non Sequenced	506 (46.0)	154 (30.0)	352 (60.0)	
Death, n (%)	165 (15.0)	88 (17.2)	77 (13.1)	0.074

BAME: Black, Asian and Minority Ethnic; BMI: Body Mass Index; HIV: human immunodeficiency virus; IMD: Index of Multiple Deprivation

Hospital mortality was 20.8% amongst 307 patients who did not receive steroids and 12.7% amongst 793 patients who received steroids. For patients who received ≤ 3 days of steroids, 17.2% died in hospital compared to 13.1% who died in hospital for patients who received > 3days of steroids. A higher mortality rate for patients who received > 10 days of steroids (24.6%) compared to patients who received ≤ 10 days of steroids (13.7%) was observed (Table1).

Comparing patient characteristics between patients who had ≤ 3 days of steroids and who had > 3 days steroids (Table1), we found that patients who had steroids for > 3 days were less likely to be of BAME ethnicity (38.2% vs. 44.6%, p = 0.035), had more obesity (37.6% vs. 30.2%, p = 0.003), had more hypertension (39.4% vs. 32.4%, p = 0.019), a higher proportion with solid organ transplatation (3.6% vs. 1.0%, p = 0.008), higher use of tocilizumab (2.0% vs. 0%, p = 0.003), and had much more Alpha variant due to the emergence of Alpha in wave two (26.9% vs. 10.3%).

### Cox proportional hazard model for the outcome of mortality

The Cox proportional hazard models showed significant protective effect of steroids used for more than 3 days compared to less steroids (HR: 0.47 (95% CI: 0.31–0.72)) for mortality. The protective effect of steroids was consistent when using steroids as a continuous variable (HR: 0.86 (95% CI: 0.76–0.96)) (Additional file Table B).

Other variables (Table3) including age, cardiovascular co-morbidity, and human immunodeficiency virus (HIV) infection had significant associations with death. The remaining variables including sex, ethnicity, IMD, hypertension, diabetes, respiratory disease, cancer, kidney disease and transplantation, Alpha variant, obesity (BMI > 30), and tocilizumab administration were not significantly associated with the outcome in the multivariable analysis.

**Table 2:** Steroids use in the cohort before and after WHO guideline (2nd September)

	Overall	Before WHO	After WHO	P-value
n	1100	366 (33.3%)	734 (66.7%)	
Steroids days, median [Q1,Q3]	4.0 [0.0,7.0]	0.0 [0.0,1.0]	5.5 [3.0,9.0]	< 0.001
Steroids days > 0, n (%)	793 (72.1)	96 (26.2)	697 (95.0)	< 0.001
Steroids days > 3, n (%)	587 (53.4)	63 (17.2)	524 (71.4)	< 0.001
Steroids days > 10, n (%)	134 (12.2)	29 (7.9)	105 (14.3)	0.003
Tocilizumab days > 0, n (%)	12 (1.1)		12 (1.6)	0.011
Death, n (%)	165 (15.0)	88 (24.0)	77 (10.5)	< 0.001

**Table 3:** Hazard Ratios (HRs) for all the variables from the multivariate cox proportional hazards models

	Outcome: in-hospital death
	steroid use > 3 days versus ≤ 3 days model
	HR (95% CI)
Age (n = 1100)	**1.04 (1.03–1.05)**
Female (n = 476)	0.96 (0.69–1.33)
Ethnicity BAME vs. White (n = 453 vs. 436)	0.99 (0.68–1.45)
Ethnicity Unknown vs. White (n = 211 vs. 436)	0.75 (0.47–1.2)
IMD Quintile (n = 1100)	1.11 (0.94–1.3)
Cardiovascular (n = 286)	**1.53 (1.09–2.15)**
Hypertension (n = 397)	0.89 (0.62–1.27)
Diabetes (n = 316)	1.19 (0.83–1.69)
Chronic respiratory disease (n = 190)	1.17 (0.79–1.74)
Cancer (n = 57)	**1.75 (1.04–2.96)**
Kidney disease (n = 135)	0.73 (0.46–1.15)
HIV infection (n = 30)	**2.21 (1.03–4.75)**
Transplantation (n = 26)	2.01 (0.74–5.5)
Alpha vs. non-Alpha (n = 211 vs. 383)	0.91 (0.54–1.53)
Non Sequenced vs. non-Alpha (n = 506 vs. 383)	1.05 (0.67–1.63)
BMI > 30 vs. BMI ≤ 30 (n = 376 vs. 524)	0.67 (0.43–1.05)
BMI Unknown vs. BMI ≤ 30 (n = 200 vs. 524)	**2.35 (1.64–3.38)**
Tocilizumab or sarilumab (n = 12)	1.93 (0.59–6.33)
Steroid days* > 3 versus ≤ 3 (reference) (n = 587 vs. 513)	**0.47 (0.31–0.72)**

*Steroid days used in the death model were cumulative days from admission to discharge or death. BAME: Black, Asian and Minority Ethnic; IMD: Index of Multiple Deprivation; HIV: human immunodeficiency virus; BMI: Body Mass Index

## Discussion

This study provides evidence for real-world effectiveness of steroids in reducing death amongst severe COVID-19 patients. The protective effect-size of treatment with steroids was similar to that reported in the RECOVERY clinical trial [3] for a comparable group of patients defined by receipt of oxygen therapy. This adds to the evidence base for a clinical benefit of steroid treatment in COVID-19.

We adjusted for potential confounders (e.g. age, sex, ethnicity, comorbidities, BMI and IMD) as well as the characteristics of the virus (Alpha variant) and another treatment (tocilizumab) with the effect of steroids remaining statistically significant. Undoubtedly, we are unable to adjust for all confounders, including the vaccination status, other co-treatments and improvements introduced around the time of steroids e.g. thromboprophylaxis and proning which might compromise the practical use of the study findings even though the protective effects of steroids were significantly protective in the model. Vaccination could be a big confounder which was started from December 2020 and by the end of the study (17th May 2021), most of the adults had received one dose of vaccination. Regarding other co-treatments, during most of the study period, other drug therapeutics were not routinely deployed, and the effect size of newer treatments like tocilizumab were much less than steroids in clinical trials. No other SARS-CoV-2 variants that have been associated with altered severity of disease were circulating in our population during the study period.

It is notable the study was done in an institution that had good overall comparative NHS outcomes and an standardized mortality ratio (SMR) of 0.5 in ICU patients, with guidelines and practice recommending longer courses of steroids for severe patients. Over 80% of the > 10 steroid-days group were treated deliberately with long steroids and the remaining were on long term steroids as therapeutic immunomodulation for other conditions. Whether longer course of steroids has an additional benefit is not known.

Longer durations of steroids have not been systematically studied and might increase the risk/rate of adverse events, including delayed viral clearance [13]. Some studies are identifying other potential adverse events associated with steroids such as invasive mould infections including aspergillosis and mucormycosis [14], with work ongoing to assess the effect of steroids on risk of bloodstream infection [15].

In this study we investigated the association of steroid days with outcomes, however our analyses are agnostic to the dose of steroids used. There may be reasons why duration of steroid treatment mediates effects on outcomes independently of cumulative dose, for instance if a sustained period of immunosuppression is needed to prevent immune-mediated inflammation. In addition, as this study is retrospective and observational the link between steroids and the outcome is only an association and causality should not be inferred.

Many other studies on the real-world effectiveness of steroids have failed to reproduce the findings of clinical trials. Partly, this may be due to small sample size, heterogeneity of treatment and non-treatment groups, and incorrectly testing associations on individuals not expected to benefit, i.e. cases without evidence of hypoxia. Our study benefits from a wide time period for inclusion, allowing us to capture the changing treatment landscape before steroid use in COVID-19 was standardised in line with national and international guidelines. Additionally our adjustment accounts for many baseline variables which have previously been associated with severe outcomes. The validity of our analyses is supported by the findings that variables previously associated with severity, such as age and cardiovascular comorbidity retain significance in our modelling.

Other studies have found the Alpha variant of SARS-CoV-2 to be associated with severe disease, especially mortality [16–18] and hypoxia on admission [19]. However, another study in hospitalised patients did not find such an association [20]. To our knowledge, no studies on the severity of the alpha variant adjusted for newly introduced therapeutics. Interestingly, the association of alpha variant with severe disease as measured by mortality was not found in this study. This is in contrast to our initial findings in the same dataset that the Alpha variant was associated with severity as measured by hypoxia on admission [19]. It may be that severity of the alpha variant is ameliorated by efficacious treatment of hospitalised patients. This may be especially true as during the second wave steroid treatment had been introduced and standardised as the alpha variant emerged. This would also explain the disparity between findings of other published studies, with the only other study of variant status and death in hospitalised patients not finding an association.

Limitations of this study might include potential bias for patients who did not have a chance to receive steroids or received very short steroids because they were very severe and died soon after admission. This is an issue that is intractable with retrospective study, and we attempt to address this by excluding those who died in the first 24h after admission. Another limitation is that the choice of cut-offs for the steroid treatment days were chosen according to data from RECOVERY trial, our local recommendations, and WHO guidelines rather than pharmacological effect of steroids treatment in COVID-19.

## Conclusion

The protective effect of steroids in severe COVID-19 seen in our cohort is similar to that seen in clinical trials, confirming the real world effectiveness.

## Electronic supplementary material

Below is the link to the electronic supplementary material.

Supplementary Material 1

## Data Availability

The data that support the findings of this study are available from Guy’s and St Thomas’ NHS Foundation Trust (GSTT) but restrictions apply to the availability of these data, which were used under license for the current study, and so are not publicly available. Data are however available from the authors upon reasonable request and with permission of GSTT.

## List of abbreviations

ICU: Intensive Care Unit.
COVID-19: Coronavirus Disease 2019.
GSTT: Guy’s and St Thomas’ NHS Foundation Trust.
EPR: Electronic Patient Record.
IMD: Index of Multiple Deprivation.
SD: Standard Deviation.
IQR: Interquartile Range.
BMI: Body Mass Index.
HIV: Human Immunodeficiency Virus.
HR: Hazard Ratio.
