# Supplementary material for: Real-world effectiveness of steroids in severe COVID-19: a retrospective cohort study
Source: BMC Infect Dis. 2022 Oct 5;22:776. doi: 10.1186/s12879-022-07750-3 (PMC9533997; doi:10.1186/s12879-022-07750-3)
Supplement: Supplementary file 1 — Supplementary Material 1 [file 12879_2022_7750_MOESM1_ESM.docx]

**Additional file**

**Table A: Risk factors for severe disease from COVID-19 - lIterature review**

| **Risk factor** | **References** | **Comment on inclusion in multivariable model** |
| --- | --- | --- |
| Age | [[1–5]](https://paperpile.com/c/WKOrCl/4Dzf+iJdm+k0WU+ktuu+Friw) | Included as a continuous variable. |
| Sex | [[2,5]](https://paperpile.com/c/WKOrCl/iJdm+Friw) | Included as binary variable. |
| Co-morbiidities   1. Cancer 2. Chronic kidney disease 3. Chronic lung disease 4. Dementia 5. DIabetes 6. Cardiac disease 7. HIV 8. Immunocompromise 9. Liver disease 10. Obesity 11. Pregnancy 12. Transplant 13. Stroke 14. Frailty | 1. [[2,3,5–7]](https://paperpile.com/c/WKOrCl/7SHh+3dqq+k0WU+iJdm+Friw) 2. [[2,4,6]](https://paperpile.com/c/WKOrCl/7SHh+ktuu+iJdm) 3. [[2,3,5,6]](https://paperpile.com/c/WKOrCl/7SHh+k0WU+iJdm+Friw) 4. [[5,6]](https://paperpile.com/c/WKOrCl/7SHh+Friw) 5. [[2,3,6]](https://paperpile.com/c/WKOrCl/7SHh+k0WU+iJdm) 6. [[2,4–6]](https://paperpile.com/c/WKOrCl/7SHh+ktuu+iJdm+Friw) 7. [[6,8]](https://paperpile.com/c/WKOrCl/7SHh+OmoT) 8. [[2,6]](https://paperpile.com/c/WKOrCl/7SHh+iJdm) 9. [[2,5,6]](https://paperpile.com/c/WKOrCl/7SHh+iJdm+Friw) 10. [[4,6,9–11]](https://paperpile.com/c/WKOrCl/7SHh+aiyX+AfTS+rdRD+ktuu) 11. [[6,12]](https://paperpile.com/c/WKOrCl/7SHh+8t0n) 12. [[2,6]](https://paperpile.com/c/WKOrCl/7SHh+iJdm) 13. [[2,6]](https://paperpile.com/c/WKOrCl/7SHh+iJdm) 14. [[13–15]](https://paperpile.com/c/WKOrCl/0azk+yFnI+NQ6w) | 1. Included 2. Included 3. Included 4. Poorly coded in our dataset so not included. 5. Included 6. Included 7. Included 8. Poorly coded in our dataset so not included. 9. Poorly coded in our dataset so not included 10. Included 11. Small number of pregnant individuals in our dataset so not included 12. Included 13. Included with cardiovascular disease 14. Poorly coded in second wave |
| Ethnicity | [[2,5,16,17]](https://paperpile.com/c/WKOrCl/iJdm+Friw+mz42+byqJ) | Evidence suggests individuals of non-White ethnicities are at increased risk in multiple territories. Included as categorical variable. |
| Socioeconomic background | [[18,19]](https://paperpile.com/c/WKOrCl/ZhZX+qiVk) | These data suggest risk posed by certain ethnicity may be related to socioeconomic background.  For simplicity we included only ethnicity variable. |
| Variant status | [[20–22]](https://paperpile.com/c/WKOrCl/tEk7+Ngpx+nAhT) | Alpha variant associated with increased mortality in population level studies, but not in single hospitalised study (full discussion in main text) |

**REFERENCES**

1 [Richardson S, Hirsch JS, Narasimhan M, *et al.* Presenting Characteristics, Comorbidities, and Outcomes Among 5700 Patients Hospitalized With COVID-19 in the New York City Area. *JAMA* 2020;**323**:2052–9.](http://paperpile.com/b/WKOrCl/4Dzf)

2 [Williamson EJ, Walker AJ, Bhaskaran K, *et al.* Factors associated with COVID-19-related death using OpenSAFELY. *Nature* 2020;**584**:430–6.](http://paperpile.com/b/WKOrCl/iJdm)

3 [Wu Z, McGoogan JM. Characteristics of and Important Lessons From the Coronavirus Disease 2019 (COVID-19) Outbreak in China: Summary of a Report of 72 314 Cases From the Chinese Center for Disease Control and Prevention. *JAMA* 2020;**323**:1239–42.](http://paperpile.com/b/WKOrCl/k0WU)

4 [Petrilli CM, Jones SA, Yang J, *et al.* Factors associated with hospital admission and critical illness among 5279 people with coronavirus disease 2019 in New York City: prospective cohort study. *BMJ* 2020;**369**:m1966.](http://paperpile.com/b/WKOrCl/ktuu)

5 [Harrison SL, Fazio-Eynullayeva E, Lane DA, *et al.* Comorbidities associated with mortality in 31,461 adults with COVID-19 in the United States: A federated electronic medical record analysis. *PLoS Med* 2020;**17**:e1003321.](http://paperpile.com/b/WKOrCl/Friw)

6 [Know WYNT. People with Certain Medical Conditions.](http://paperpile.com/b/WKOrCl/7SHh)<https://www.cdc.gov/coronavirus/2019-ncov/need-extra-precautions/people-with-medical-conditions.html>

7 [Dai M, Liu D, Liu M, *et al.* Patients with Cancer Appear More Vulnerable to SARS-CoV-2: A Multicenter Study during the COVID-19 Outbreak. *Cancer Discov* 2020;**10**:783–91.](http://paperpile.com/b/WKOrCl/3dqq)

8 [Geretti AM, Stockdale AJ, Kelly SH, *et al.* Outcomes of COVID-19 related hospitalization among people with HIV in the ISARIC WHO Clinical Characterization Protocol (UK): a prospective observational study. *Clin Infect Dis* Published Online First: 23 October 2020. doi:](http://paperpile.com/b/WKOrCl/OmoT)[10.1093/cid/ciaa1605](http://dx.doi.org/10.1093/cid/ciaa1605)

9 [Lighter J, Phillips M, Hochman S, *et al.* Obesity in Patients Younger Than 60 Years Is a Risk Factor for COVID-19 Hospital Admission. *Clin Infect Dis* 2020;**71**:896–7.](http://paperpile.com/b/WKOrCl/aiyX)

10  [Tartof SY, Qian L, Hong V, *et al.* Obesity and Mortality Among Patients Diagnosed With COVID-19: Results From an Integrated Health Care Organization. *Ann Intern Med* 2020;**173**:773–81.](http://paperpile.com/b/WKOrCl/AfTS)

11  [Kompaniyets L, Goodman AB, Belay B, *et al.* Body Mass Index and Risk for COVID-19-Related Hospitalization, Intensive Care Unit Admission, Invasive Mechanical Ventilation, and Death - United States, March-December 2020. *MMWR Morb Mortal Wkly Rep* 2021;**70**:355–61.](http://paperpile.com/b/WKOrCl/rdRD)

12  [Allotey J, Stallings E, Bonet M, *et al.* Clinical manifestations, risk factors, and maternal and perinatal outcomes of coronavirus disease 2019 in pregnancy: living systematic review and meta-analysis. *BMJ* 2020;**370**:m3320.](http://paperpile.com/b/WKOrCl/8t0n)

13  [Dumitrascu F, Branje KE, Hladkowicz ES, *et al.* Association of frailty with outcomes in individuals with COVID-19: A living review and meta-analysis. *J Am Geriatr Soc* 2021;**69**:2419–29.](http://paperpile.com/b/WKOrCl/0azk)

14  [Kastora S, Kounidas G, Perrott S, *et al.* Clinical frailty scale as a point of care prognostic indicator of mortality in COVID-19: a systematic review and meta-analysis. *EClinicalMedicine* 2021;**36**:100896.](http://paperpile.com/b/WKOrCl/yFnI)

15  [Yang Y, Luo K, Jiang Y, *et al.* The Impact of Frailty on COVID-19 Outcomes: A Systematic Review and Meta-analysis of 16 Cohort Studies. *J Nutr Health Aging* 2021;**25**:702–9.](http://paperpile.com/b/WKOrCl/NQ6w)

16  [Price-Haywood EG, Burton J, Fort D, *et al.* Hospitalization and Mortality among Black Patients and White Patients with Covid-19. *N Engl J Med* 2020;**382**:2534–43.](http://paperpile.com/b/WKOrCl/mz42)

17  [Gold JAW, Rossen LM, Ahmad FB, *et al.* Race, Ethnicity, and Age Trends in Persons Who Died from COVID-19 - United States, May-August 2020. *MMWR Morb Mortal Wkly Rep* 2020;**69**:1517–21.](http://paperpile.com/b/WKOrCl/byqJ)

18  [Kabarriti R, Brodin NP, Maron MI, *et al.* Association of Race and Ethnicity With Comorbidities and Survival Among Patients With COVID-19 at an Urban Medical Center in New York. *JAMA Netw Open* 2020;**3**:e2019795.](http://paperpile.com/b/WKOrCl/ZhZX)

19  [Muñoz-Price LS, Nattinger AB, Rivera F, *et al.* Racial Disparities in Incidence and Outcomes Among Patients With COVID-19. *JAMA Netw Open* 2020;**3**:e2021892.](http://paperpile.com/b/WKOrCl/qiVk)

20  [Davies NG, Jarvis CI, CMMID COVID-19 Working Group, *et al.* Increased mortality in community-tested cases of SARS-CoV-2 lineage B.1.1.7. *Nature* 2021;**593**:270–4.](http://paperpile.com/b/WKOrCl/tEk7)

21  [Frampton D, Rampling T, Cross A, *et al.* Genomic characteristics and clinical effect of the emergent SARS-CoV-2 B.1.1.7 lineage in London, UK: a whole-genome sequencing and hospital-based cohort study. *Lancet Infect Dis* Published Online First: 12 April 2021. doi:](http://paperpile.com/b/WKOrCl/Ngpx)[10.1016/S1473-3099(21)00170-5](http://dx.doi.org/10.1016/S1473-3099(21)00170-5)

22  [Challen R, Brooks-Pollock E, Read JM, *et al.* Risk of mortality in patients infected with SARS-CoV-2 variant of concern 202012/1: matched cohort study. *BMJ* 2021;**372**:n579.](http://paperpile.com/b/WKOrCl/nAhT)

**Table B: Hazard Ratios (HRs) for all the variables from the multivariate cox proportional hazards models with outcomes as death and with duration of steroid as a continuous variable (log transformation applied)**

|  | **Death** |  |
| --- | --- | --- |
|  | HR | 95% CI |
| Age | 1.04 | **1.03-1.06** |
| Female | 0.97 | 0.7-1.34 |
| Ethnicity BAME vs White | 1.04 | 0.72-1.52 |
| Ethnicity Unknown vs White | 0.72 | 0.44-1.17 |
| IMD Quintile | 1.09 | 0.93-1.28 |
| Cardiovascular | 1.54 | **1.1-2.17** |
| Hypertension | 0.84 | 0.58-1.21 |
| Diabetes | 1.18 | 0.83-1.69 |
| Chronic respiratory disease | 1.16 | 0.78-1.73 |
| Cancer | 1.69 | **1.0-2.84** |
| Kidney disease | 0.71 | 0.45-1.13 |
| HIV infection | 2.49 | **1.16-5.33** |
| Transplantation | 1.6 | 0.55-4.71 |
| Alpha vs non-Alpha | 0.8 | 0.47-1.37 |
| nonSequenced vs non-Alpha | 0.95 | 0.61-1.49 |
| BMI>30 vs BMI≤30 | 0.65 | 0.41-1.03 |
| BMI Unknown vs BMI≤30 | 2.31 | **1.61-3.31** |
| Tocilizumab or sarilumab+ | 1.93 | 0.59-6.3 |
| log(steroid days*+1) | 0.86 | **0.76-0.96** |

*Steroid-days used in the death model were cumulative days from admission to discharge or death.

+ tocilizumab and sarilumab were only used in ICU.

BAME: Black, Asian and Minority Ethnic; IMD: Index of Multiple Deprivation; HIV: human immunodeficiency virus; BMI: Body Mass Index.
